# *cis*-Bis{1-[(4-nitro­phen­yl)diazen­yl]-2-naphtholato}dipyridine­nickel(II)^1^

**DOI:** 10.1107/S1600536808040476

**Published:** 2008-12-06

**Authors:** Lorenzo do Canto Visentin, Carlos Alberto Lombardi Filgueiras, Jairo Bordinhão, Leonardo da Cunha Ferreira

**Affiliations:** a Instituto de Química, Universidade Federal do Rio de Janeiro, Caixa Postal 68563, 21949-900 Rio de Janeiro, RJ, Brazil

## Abstract

In the title compound, [Ni(C_16_H_10_N_3_O_3_)_2_(C_5_H_5_N)_2_], the Ni^II^ cation is in a distorted octa­hedral NiN_4_O_2_ coordination by two independent bidentate 1-[(4-nitro­phen­yl)diazen­yl]-2-naph­thol­ate anions and two pyridine ligands. C—H⋯O inter­actions between aromatic rings and the O atoms of the nitro substituents build up a two-dimensional supra­molecular arrangement parallel to (100).

## Related literature

For background on metal azo complexes, see: Carella *et al.* (2007 ▶); Kulikovska *et al.* (2007 ▶); Patnaik *et al.* (2007 ▶); Leng *et al.* (2001 ▶). For bond lengths, see: Abildgaard *et al.* (2006 ▶). For hydrogen bonds, see: Jeffrey & Saenger (1991 ▶).
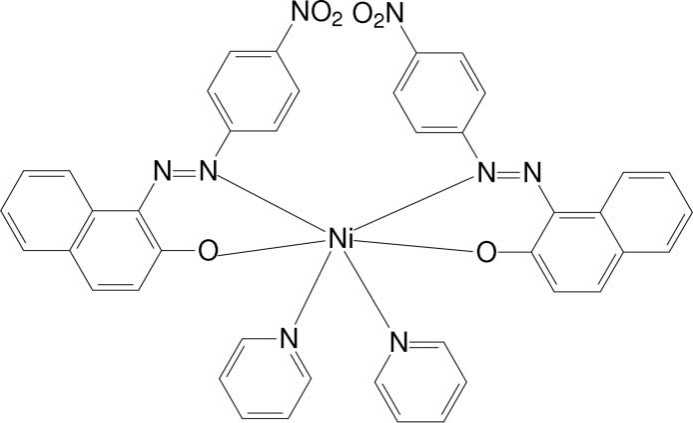


## Experimental

### 

#### Crystal data

[Ni(C_16_H_10_N_3_O_3_)_2_(C_5_H_5_N)_2_]
*M* *_r_* = 801.45
Monoclinic, 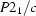

*a* = 11.719 (2) Å
*b* = 18.885 (4) Å
*c* = 16.922 (3) Å
β = 93.04 (3)°
*V* = 3740.0 (13) Å^3^
*Z* = 4
Mo *K*α radiation
μ = 0.58 mm^−1^
*T* = 295 (2) K
0.37 × 0.18 × 0.15 mm

#### Data collection

Nonius KappaCCD diffractometer
Absorption correction: multi-scan (*SADABS*; Sheldrick, 2004 ▶) *T*_min_ = 0.814, *T*_max_ = 0.918
64998 measured reflections
6566 independent reflections
4859 reflections with *I* > 2σ(*I*)
*R*_int_ = 0.076

#### Refinement

*R*[*F* ^2^ > 2σ(*F* ^2^)] = 0.039
*wR*(*F* ^2^) = 0.090
*S* = 1.05
6566 reflections
514 parameters
H-atom parameters constrained
Δρ_max_ = 0.23 e Å^−3^
Δρ_min_ = −0.24 e Å^−3^

### 

Data collection: *COLLECT* (Nonius, 1998 ▶); cell refinement: *PHICHI* (Duisenberg *et al*., 2000 ▶); data reduction: *EVALCCD* (Duisenberg *et al*., 2003 ▶); program(s) used to solve structure: *SHELXS97* (Sheldrick, 2008 ▶); program(s) used to refine structure: *SHELXL97* (Sheldrick, 2008 ▶); molecular graphics: *ORTEP-3 for Windows* (Farrugia, 1997 ▶); software used to prepare material for publication: *PLATON* (Spek, 2003 ▶) and *WinGX* (Farrugia, 1999 ▶).

## Supplementary Material

Crystal structure: contains datablocks global, I. DOI: 10.1107/S1600536808040476/wm2206sup1.cif

Structure factors: contains datablocks I. DOI: 10.1107/S1600536808040476/wm2206Isup2.hkl

Additional supplementary materials:  crystallographic information; 3D view; checkCIF report

## Figures and Tables

**Table 1:** Selected bond lengths (Å)

Ni1—N1	2.168 (2)
Ni1—N4	2.143 (2)
Ni1—N7	2.121 (2)
Ni1—N8	2.122 (2)
Ni1—O1	2.0116 (16)
Ni1—O4	2.0161 (17)

**Table 2:** Hydrogen-bond geometry (Å, °)

*D*—H⋯*A*	*D*—H	H⋯*A*	*D*⋯*A*	*D*—H⋯*A*
C36—H36⋯O2^i^	0.93	2.38	3.124 (5)	137
C41—H41⋯O6^ii^	0.93	2.53	3.205 (4)	130

Symmetry codes: (i) 
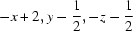
; (ii) 
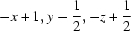
.

**Fig. 1.**
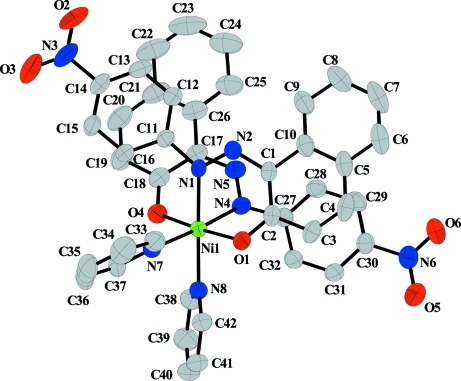
ORTEP plot of the title molecule, (I). Displacement ellipsoids are drawn at the 50% probability level.

**Fig. 2.**
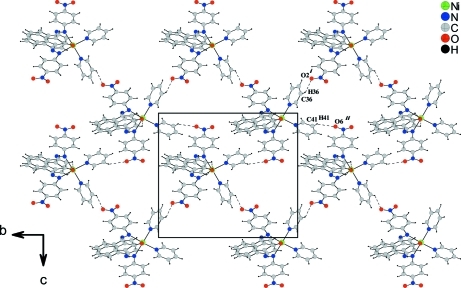
View of the intermolecular H-bonding interactions and the formation of the two-dimensional arrangement. [Symmetry codes: (i) -x + 2, y - 1/2, -z - 1/2; (ii) -x + 1, y - 1/2, -z + 1/2.]

## supplementary crystallographic information

### Comment

Metal complexes with azo ligands show interesting chemical and physical properties and are of interest as new materials, for example in bioinorganic and coordination chemistry, as well as in biological systems which can lead to the development of new products with specific properties (Carella *et al.*, 2007; Kulikovska *et al.*, 2007; Patnaik *et al.*, 2007; Leng *et al.*, 2001). In this work the structure of the title molecule, [Ni(C_16_H_10_N_3_O_3_)_2_(C_5_H_5_N)_2_], (I) is reported.

Fig. 1 shows the molecular structure of compound (I). The Ni^II^ cation is octahedrally coordinated by four N and two O atoms with only slight distortion from the ideal coordination geometry. The two independent 1-[(4-nitrophenyl)diazenyl]-2-naphtholate ligands are bidentate and provide each one N atom from the azene moiety and one naphtolate O atom. The coordination is completed by the two pyridine N atoms. The Ni—N and Ni—O distances (Table 1) are in the typical ranges and like all other interatomic distances are in good agreement with literature data (Abildgaard *et al.*, 2006).

The crystal packing is accomplished by two non-classical intermolecular C—H···O hydrogen bonds (Jeffrey & Saenger, 1991), forming a two-dimensional arrangement parallel to (100) (Fig. 2).

### Experimental

To a mixture of 10.0 ml of MeOH and 10.0 ml of pyridine, 0.058 g (0.2 mmol) of 4-nitrophenylazo-2-naphthole was added with continuous stirring at room temperature. After stirring for 20 min, 0.025 g (0.1 mmol) of Ni(II) acetate were added. Stirring was maintained for 24 h. The solution was filtered off and red crystals of (I) with a block habit and up to 0.4 mm maximum size were obtained by slow evaporation of the mixture at room temperature. Melting point: 473 K; C, H, N analysis (%): calc., C, 62.94; H, 3.77; N, 13.98; found, C, 64.11; H, 3.91; N, 12.61.

### Refinement

The H atoms of the naphthyl, pyridine and phenyl rings were fixed geometrically at a distance of 0.93 Å and were refined in the riding model approximation with *U*_iso_(H) = 1.2×*U*_eq_ of the parent C atom.

### Crystal data

**Table:**

[Ni(C_16_H_10_N_3_O_3_)_2_(C_5_H_5_N)_2_]	*F*(000) = 1656
*M**_r_* = 801.45	*D*_x_ = 1.423 Mg m^−^^3^
Monoclinic, *P*2_1_/*c*	Melting point: 473 K
Hall symbol: -P 2ybc	Mo *K*α radiation, λ = 0.71073 Å
*a* = 11.719 (2) Å	Cell parameters from 217 reflections
*b* = 18.885 (4) Å	θ = 1.0–27.5°
*c* = 16.922 (3) Å	µ = 0.58 mm^−^^1^
β = 93.04 (3)°	*T* = 295 K
*V* = 3740.0 (13) Å^3^	Block, red
*Z* = 4	0.37 × 0.18 × 0.15 mm

### Data collection

**Table:**

Nonius KappaCCD diffractometer	6566 independent reflections
Radiation source: fine-focus sealed tube	4859 reflections with *I* > 2σ(*I*)
graphite	*R*_int_ = 0.076
φ and ω scans	θ_max_ = 25.0°, θ_min_ = 2.8°
Absorption correction: multi-scan (*SADABS*; Sheldrick, 2004)	*h* = −13→13
*T*_min_ = 0.814, *T*_max_ = 0.918	*k* = −22→22
64998 measured reflections	*l* = −20→20

### Refinement

**Table:**

Refinement on *F*^2^	Primary atom site location: structure-invariant direct methods
Least-squares matrix: full	Secondary atom site location: difference Fourier map
*R*[*F*^2^ > 2σ(*F*^2^)] = 0.039	Hydrogen site location: inferred from neighbouring sites
*wR*(*F*^2^) = 0.090	H-atom parameters constrained
*S* = 1.05	*w* = 1/[σ^2^(*F*_o_^2^) + (0.0357*P*)^2^ + 1.6025*P*] where *P* = (*F*_o_^2^ + 2*F*_c_^2^)/3
6566 reflections	(Δ/σ)_max_ < 0.001
514 parameters	Δρ_max_ = 0.23 e Å^−^^3^
0 restraints	Δρ_min_ = −0.24 e Å^−^^3^

### Special details

**Table:**

Experimental. Least-squares planes (*x*,*y*,*z* in crystal coordinates) and deviations from them (* indicates atom used to define plane)5.9841 (0.0107) *x* + 0.5948 (0.0176) *y* + 14.0605 (0.0119) *z* = 4.0378 (0.0057)* 0.0370 (0.0010) N1 * -0.0652 (0.0015) N2 * 0.0496 (0.0015) C1 * -0.0087 (0.0015) C2 * -0.0127 (0.0010) O1Rms deviation of fitted atoms = 0.0408- 0.4462 (0.0069) *x* + 8.8584 (0.0115) *y* + 14.9440 (0.0093) *z* = 1.2966 (0.0053)Angle to previous plane (with approximate e.s.d.) = 41.11 (0.07)* 0.0666 (0.0009) O1 * -0.0633 (0.0009) N1 * 0.0616 (0.0008) O4 * -0.0623 (0.0009) N8 * -0.0026 (0.0008) Ni1Rms deviation of fitted atoms = 0.05686.2807 (0.0104) *x* - 6.1779 (0.0201) *y* + 12.6704 (0.0108) *z* = 5.5919 (0.0111)Angle to previous plane (with approximate e.s.d.) = 59.14 (0.06)* -0.0118 (0.0011) O4 * 0.0423 (0.0016) C18 * -0.0623 (0.0015) C17 * 0.0507 (0.0015) N5 * -0.0189 (0.0010) N4Rms deviation of fitted atoms = 0.0418- 0.0859 (0.0070) *x* + 16.1355 (0.0106) *y* - 8.7731 (0.0106) *z* = 1.4081 (0.0053)Angle to previous plane (with approximate e.s.d.) = 46.54 (0.09)* 0.0058 (0.0009) O4 * 0.0056 (0.0010) N4 * 0.0063 (0.0009) O1 * 0.0060 (0.0009) N7 * -0.0236 (0.0008) Ni1Rms deviation of fitted atoms = 0.01186.5874 (0.0126) *x* + 14.2991 (0.0164) *y* - 6.1291 (0.0186) *z* = 6.0049 (0.0088)Angle to previous plane (with approximate e.s.d.) = 35.34 (0.09)* 0.0044 (0.0017) N7 * -0.0069 (0.0020) C33 * 0.0029 (0.0023) C34 * 0.0033 (0.0025) C35 * -0.0056 (0.0023) C36 * 0.0019 (0.0019) C37Rms deviation of fitted atoms = 0.00456.8092 (0.0117) *x* - 4.2913 (0.0191) *y* - 13.7284 (0.0134) *z* = 3.6963 (0.0099)Angle to previous plane (with approximate e.s.d.) = 65.50 (0.09)* 0.0087 (0.0016) N8 * -0.0004 (0.0019) C38 * -0.0074 (0.0021) C39 * 0.0071 (0.0020) C40 * 0.0011 (0.0019) C41 * -0.0092 (0.0018) C42Rms deviation of fitted atoms = 0.00677.6103 (0.0099) *x* - 1.0403 (0.0194) *y* + 12.2331 (0.0135) *z* = 7.5132 (0.0060)Angle to previous plane (with approximate e.s.d.) = 78.29 (0.07)* -0.0081 (0.0017) C27 * 0.0104 (0.0018) C28 * -0.0020 (0.0018) C29 * -0.0088 (0.0018) C30 * 0.0109 (0.0019) C31 * -0.0025 (0.0019) C32Rms deviation of fitted atoms = 0.00796.0882 (0.0095) *x* - 3.0287 (0.0105) *y* + 13.7154 (0.0112) *z* = 6.1985 (0.0098)Angle to previous plane (with approximate e.s.d.) = 10.66 (0.13)* 0.0072 (0.0020) C17 * -0.1226 (0.0020) C18 * 0.0188 (0.0022) C19 * 0.0637 (0.0024) C20 * 0.0353 (0.0026) C21 * -0.0368 (0.0028) C22 * -0.0666 (0.0033) C23 * -0.0092 (0.0031) C24 * 0.0452 (0.0026) C25 * 0.0652 (0.0024) C26Rms deviation of fitted atoms = 0.05746.0989 (0.0089) *x* - 1.3127 (0.0101) *y* + 13.9139 (0.0107) *z* = 3.6881 (0.0050)Angle to previous plane (with approximate e.s.d.) = 5.25 (0.11)* -0.0019 (0.0019) C1 * 0.0685 (0.0019) C2 * -0.0202 (0.0021) C3 * -0.0339 (0.0022) C4 * -0.0136 (0.0024) C5 * 0.0221 (0.0025) C6 * 0.0381 (0.0028) C7 * -0.0039 (0.0027) C8 * -0.0255 (0.0024) C9 * -0.0297 (0.0023) C10Rms deviation of fitted atoms = 0.03157.6507 (0.0101) *x* - 0.7993 (0.0215) *y* + 12.1938 (0.0139) *z* = 4.9408 (0.0114)Angle to previous plane (with approximate e.s.d.) = 9.47 (0.13)* 0.0100 (0.0017) C11 * -0.0050 (0.0019) C12 * -0.0055 (0.0021) C13 * 0.0110 (0.0019) C14 * -0.0058 (0.0018) C15 * -0.0048 (0.0018) C16Rms deviation of fitted atoms = 0.0075
Geometry. All e.s.d.'s (except the e.s.d. in the dihedral angle between two l.s. planes) are estimated using the full covariance matrix. The cell e.s.d.'s are taken into account individually in the estimation of e.s.d.'s in distances, angles and torsion angles; correlations between e.s.d.'s in cell parameters are only used when they are defined by crystal symmetry. An approximate (isotropic) treatment of cell e.s.d.'s is used for estimating e.s.d.'s involving l.s. planes.
Refinement. Refinement of *F*^2^ against ALL reflections. The weighted *R*-factor *wR* and goodness of fit *S* are based on *F*^2^, conventional *R*-factors *R* are based on *F*, with *F* set to zero for negative *F*^2^. The threshold expression of *F*^2^ > σ(*F*^2^) is used only for calculating *R*-factors(gt) *etc*. and is not relevant to the choice of reflections for refinement. *R*-factors based on *F*^2^ are statistically about twice as large as those based on *F*, and *R*- factors based on ALL data will be even larger.

### Fractional atomic coordinates and isotropic or equivalent isotropic displacement parameters (Å^2^)

**Table:**

	*x*	*y*	*z*	*U*_iso_*/*U*_eq_	
Ni1	0.73654 (2)	0.112500 (16)	0.041889 (18)	0.03358 (10)	
N1	0.71063 (15)	0.21142 (10)	−0.02158 (11)	0.0336 (4)	
N2	0.63714 (16)	0.25763 (10)	0.00047 (11)	0.0370 (5)	
N3	1.0357 (2)	0.33257 (17)	−0.21680 (16)	0.0701 (8)	
N4	0.75644 (16)	0.17270 (10)	0.14908 (11)	0.0379 (5)	
N5	0.81962 (17)	0.22968 (11)	0.15104 (12)	0.0401 (5)	
N6	0.4055 (2)	0.16963 (14)	0.36697 (13)	0.0531 (6)	
N7	0.73012 (17)	0.05551 (10)	−0.06623 (12)	0.0406 (5)	
N8	0.74895 (17)	0.01201 (10)	0.09784 (12)	0.0386 (5)	
C1	0.54975 (19)	0.23983 (13)	0.04658 (13)	0.0358 (5)	
C2	0.51196 (19)	0.16895 (14)	0.06152 (14)	0.0373 (6)	
C3	0.4060 (2)	0.16159 (16)	0.10089 (15)	0.0471 (6)	
H3	0.3751	0.1166	0.1067	0.056*	
C4	0.3506 (2)	0.21795 (17)	0.12949 (16)	0.0543 (8)	
H4	0.2844	0.2103	0.1561	0.065*	
C5	0.3901 (2)	0.28914 (16)	0.12038 (15)	0.0480 (7)	
C6	0.3342 (3)	0.3474 (2)	0.15296 (18)	0.0678 (9)	
H6	0.2696	0.3398	0.1815	0.081*	
C7	0.3728 (3)	0.4148 (2)	0.1435 (2)	0.0803 (11)	
H7	0.3356	0.4527	0.1662	0.096*	
C8	0.4683 (3)	0.42665 (18)	0.0997 (2)	0.0732 (10)	
H8	0.4937	0.4727	0.0920	0.088*	
C9	0.5259 (3)	0.37042 (15)	0.06765 (18)	0.0577 (8)	
H9	0.5904	0.3791	0.0394	0.069*	
C10	0.4883 (2)	0.30047 (14)	0.07722 (14)	0.0426 (6)	
C11	0.78929 (19)	0.24392 (12)	−0.07322 (13)	0.0340 (5)	
C12	0.8024 (2)	0.31680 (14)	−0.07792 (16)	0.0482 (7)	
H12	0.7574	0.3463	−0.0485	0.058*	
C13	0.8815 (3)	0.34579 (15)	−0.12567 (17)	0.0575 (8)	
H13	0.8900	0.3947	−0.1287	0.069*	
C14	0.9481 (2)	0.30173 (15)	−0.16898 (15)	0.0465 (7)	
C15	0.9355 (2)	0.22981 (14)	−0.16720 (15)	0.0451 (6)	
H15	0.9798	0.2009	−0.1978	0.054*	
C16	0.8561 (2)	0.20082 (13)	−0.11915 (15)	0.0415 (6)	
H16	0.8470	0.1519	−0.1174	0.050*	
C17	0.9075 (2)	0.23637 (13)	0.10183 (14)	0.0413 (6)	
C18	0.9558 (2)	0.17932 (14)	0.05833 (15)	0.0425 (6)	
C19	1.0637 (2)	0.19248 (16)	0.02366 (17)	0.0532 (7)	
H19	1.1029	0.1548	0.0024	0.064*	
C20	1.1091 (2)	0.25816 (18)	0.02127 (18)	0.0605 (8)	
H20	1.1771	0.2647	−0.0037	0.073*	
C21	1.0559 (2)	0.31780 (17)	0.05598 (17)	0.0571 (8)	
C22	1.0988 (3)	0.3870 (2)	0.0470 (2)	0.0810 (11)	
H22	1.1630	0.3941	0.0180	0.097*	
C23	1.0472 (4)	0.4438 (2)	0.0802 (3)	0.0974 (14)	
H23	1.0756	0.4892	0.0734	0.117*	
C24	0.9519 (4)	0.43321 (19)	0.1244 (3)	0.0902 (12)	
H24	0.9180	0.4717	0.1481	0.108*	
C25	0.9069 (3)	0.36590 (16)	0.13346 (19)	0.0663 (9)	
H25	0.8424	0.3598	0.1624	0.080*	
C26	0.9579 (2)	0.30722 (15)	0.09933 (16)	0.0495 (7)	
C27	0.6742 (2)	0.17444 (13)	0.20889 (13)	0.0376 (6)	
C28	0.6336 (2)	0.23743 (13)	0.24103 (15)	0.0418 (6)	
H28	0.6650	0.2805	0.2268	0.050*	
C29	0.5478 (2)	0.23611 (14)	0.29330 (15)	0.0449 (6)	
H29	0.5202	0.2781	0.3138	0.054*	
C30	0.5028 (2)	0.17145 (14)	0.31523 (14)	0.0416 (6)	
C31	0.5433 (2)	0.10847 (14)	0.28627 (14)	0.0463 (6)	
H31	0.5140	0.0654	0.3026	0.056*	
C32	0.6281 (2)	0.11035 (14)	0.23263 (15)	0.0466 (6)	
H32	0.6547	0.0682	0.2120	0.056*	
C33	0.6487 (2)	0.06812 (15)	−0.12249 (16)	0.0511 (7)	
H33	0.5897	0.0989	−0.1115	0.061*	
C34	0.6486 (3)	0.03716 (19)	−0.19646 (19)	0.0736 (10)	
H34	0.5913	0.0475	−0.2349	0.088*	
C35	0.7351 (3)	−0.0094 (2)	−0.2122 (2)	0.0834 (12)	
H35	0.7370	−0.0310	−0.2615	0.100*	
C36	0.8178 (3)	−0.02345 (18)	−0.1546 (2)	0.0731 (10)	
H36	0.8763	−0.0552	−0.1639	0.088*	
C37	0.8135 (2)	0.00968 (14)	−0.08311 (17)	0.0519 (7)	
H37	0.8707	0.0002	−0.0443	0.062*	
C38	0.8368 (2)	−0.00533 (14)	0.14751 (16)	0.0485 (7)	
H38	0.8893	0.0297	0.1627	0.058*	
C39	0.8535 (3)	−0.07288 (16)	0.17741 (18)	0.0595 (8)	
H39	0.9154	−0.0828	0.2123	0.071*	
C40	0.7765 (3)	−0.12535 (15)	0.15457 (18)	0.0580 (8)	
H40	0.7864	−0.1715	0.1729	0.070*	
C41	0.6855 (2)	−0.10789 (14)	0.10441 (17)	0.0512 (7)	
H41	0.6321	−0.1421	0.0884	0.061*	
C42	0.6738 (2)	−0.03930 (13)	0.07789 (16)	0.0444 (6)	
H42	0.6107	−0.0280	0.0446	0.053*	
O1	0.56484 (13)	0.11301 (9)	0.04110 (10)	0.0399 (4)	
O2	1.0358 (3)	0.39692 (16)	−0.22459 (19)	0.1256 (12)	
O3	1.1043 (2)	0.29343 (15)	−0.24720 (15)	0.0888 (8)	
O4	0.90854 (14)	0.11845 (9)	0.04779 (10)	0.0449 (4)	
O5	0.3680 (2)	0.11152 (12)	0.38569 (14)	0.0799 (7)	
O6	0.36631 (19)	0.22562 (12)	0.38958 (14)	0.0758 (7)	

### Atomic displacement parameters (Å^2^)

**Table:**

	*U*^11^	*U*^22^	*U*^33^	*U*^12^	*U*^13^	*U*^23^
Ni1	0.03349 (16)	0.03100 (16)	0.03668 (17)	−0.00179 (13)	0.00593 (12)	−0.00190 (14)
N1	0.0334 (10)	0.0355 (11)	0.0326 (11)	−0.0007 (9)	0.0084 (8)	−0.0021 (9)
N2	0.0360 (11)	0.0394 (12)	0.0358 (11)	0.0018 (9)	0.0035 (9)	−0.0009 (9)
N3	0.0730 (18)	0.082 (2)	0.0574 (16)	−0.0254 (16)	0.0229 (14)	0.0154 (15)
N4	0.0411 (11)	0.0361 (11)	0.0367 (11)	−0.0049 (9)	0.0021 (9)	0.0001 (9)
N5	0.0453 (12)	0.0389 (12)	0.0354 (11)	−0.0060 (10)	−0.0040 (9)	0.0009 (9)
N6	0.0605 (15)	0.0575 (16)	0.0422 (13)	0.0017 (13)	0.0103 (11)	−0.0041 (12)
N7	0.0450 (12)	0.0345 (11)	0.0432 (12)	−0.0056 (9)	0.0103 (10)	−0.0051 (10)
N8	0.0407 (11)	0.0347 (11)	0.0410 (12)	0.0008 (9)	0.0063 (9)	−0.0002 (9)
C1	0.0346 (13)	0.0434 (14)	0.0299 (13)	0.0023 (11)	0.0045 (10)	−0.0015 (11)
C2	0.0308 (12)	0.0500 (15)	0.0311 (13)	0.0022 (11)	0.0005 (10)	−0.0028 (11)
C3	0.0313 (13)	0.0617 (18)	0.0488 (16)	−0.0056 (12)	0.0077 (11)	0.0017 (14)
C4	0.0323 (14)	0.089 (2)	0.0428 (16)	0.0034 (14)	0.0072 (12)	−0.0034 (15)
C5	0.0369 (14)	0.0677 (19)	0.0392 (15)	0.0134 (13)	0.0006 (11)	−0.0089 (13)
C6	0.0507 (18)	0.093 (3)	0.060 (2)	0.0191 (18)	0.0077 (15)	−0.0226 (19)
C7	0.074 (2)	0.084 (3)	0.083 (3)	0.032 (2)	0.004 (2)	−0.036 (2)
C8	0.080 (2)	0.057 (2)	0.083 (2)	0.0170 (17)	0.0030 (19)	−0.0207 (18)
C9	0.0619 (18)	0.0523 (18)	0.0594 (19)	0.0119 (14)	0.0090 (15)	−0.0092 (14)
C10	0.0426 (14)	0.0510 (16)	0.0340 (14)	0.0106 (12)	−0.0011 (11)	−0.0044 (12)
C11	0.0357 (13)	0.0371 (13)	0.0291 (12)	−0.0040 (10)	0.0005 (10)	0.0022 (10)
C12	0.0582 (17)	0.0404 (15)	0.0473 (16)	−0.0031 (12)	0.0155 (13)	−0.0014 (12)
C13	0.072 (2)	0.0411 (16)	0.0603 (19)	−0.0136 (14)	0.0173 (15)	0.0090 (14)
C14	0.0456 (15)	0.0587 (18)	0.0358 (14)	−0.0105 (13)	0.0074 (12)	0.0098 (13)
C15	0.0435 (15)	0.0518 (17)	0.0413 (15)	0.0018 (12)	0.0131 (12)	0.0049 (12)
C16	0.0430 (14)	0.0362 (14)	0.0464 (15)	−0.0019 (11)	0.0128 (12)	0.0032 (12)
C17	0.0412 (14)	0.0465 (15)	0.0353 (14)	−0.0087 (12)	−0.0053 (11)	0.0041 (12)
C18	0.0353 (13)	0.0508 (17)	0.0409 (14)	−0.0035 (12)	−0.0032 (11)	0.0084 (12)
C19	0.0363 (14)	0.0658 (19)	0.0575 (18)	−0.0043 (13)	0.0024 (13)	0.0042 (15)
C20	0.0383 (15)	0.087 (2)	0.0555 (18)	−0.0191 (16)	−0.0039 (13)	0.0154 (17)
C21	0.0522 (17)	0.066 (2)	0.0515 (17)	−0.0233 (15)	−0.0131 (14)	0.0154 (15)
C22	0.074 (2)	0.080 (3)	0.087 (3)	−0.040 (2)	−0.0172 (19)	0.027 (2)
C23	0.117 (4)	0.056 (2)	0.116 (4)	−0.038 (2)	−0.020 (3)	0.020 (2)
C24	0.114 (3)	0.050 (2)	0.104 (3)	−0.021 (2)	−0.008 (3)	−0.004 (2)
C25	0.081 (2)	0.0481 (18)	0.069 (2)	−0.0153 (16)	−0.0033 (17)	−0.0007 (15)
C26	0.0538 (16)	0.0490 (17)	0.0442 (16)	−0.0140 (13)	−0.0131 (13)	0.0053 (13)
C27	0.0435 (14)	0.0403 (14)	0.0287 (12)	−0.0014 (11)	−0.0010 (10)	−0.0031 (11)
C28	0.0486 (15)	0.0366 (14)	0.0399 (15)	−0.0065 (11)	0.0010 (12)	−0.0024 (11)
C29	0.0555 (16)	0.0387 (15)	0.0405 (15)	0.0013 (12)	0.0021 (12)	−0.0106 (12)
C30	0.0461 (14)	0.0480 (16)	0.0310 (13)	0.0009 (12)	0.0032 (11)	−0.0040 (12)
C31	0.0629 (17)	0.0366 (14)	0.0404 (14)	−0.0010 (13)	0.0132 (12)	0.0038 (12)
C32	0.0624 (17)	0.0366 (14)	0.0420 (14)	0.0016 (13)	0.0129 (12)	−0.0015 (12)
C33	0.0589 (17)	0.0480 (17)	0.0466 (16)	−0.0088 (13)	0.0054 (14)	−0.0072 (13)
C34	0.080 (2)	0.090 (3)	0.0504 (19)	−0.027 (2)	−0.0005 (17)	−0.0147 (18)
C35	0.099 (3)	0.091 (3)	0.064 (2)	−0.031 (2)	0.037 (2)	−0.042 (2)
C36	0.072 (2)	0.069 (2)	0.082 (2)	−0.0109 (18)	0.037 (2)	−0.032 (2)
C37	0.0527 (16)	0.0437 (16)	0.0614 (18)	−0.0034 (13)	0.0233 (14)	−0.0109 (14)
C38	0.0496 (16)	0.0451 (16)	0.0505 (16)	0.0002 (12)	−0.0006 (13)	−0.0016 (13)
C39	0.0621 (19)	0.0571 (19)	0.0584 (19)	0.0098 (15)	−0.0056 (15)	0.0089 (15)
C40	0.074 (2)	0.0377 (16)	0.0638 (19)	0.0050 (14)	0.0153 (16)	0.0099 (14)
C41	0.0599 (17)	0.0389 (15)	0.0557 (17)	−0.0084 (13)	0.0102 (14)	0.0021 (13)
C42	0.0449 (15)	0.0391 (15)	0.0495 (16)	−0.0038 (12)	0.0060 (12)	0.0010 (12)
O1	0.0348 (9)	0.0402 (9)	0.0453 (10)	−0.0050 (8)	0.0083 (7)	−0.0032 (8)
O2	0.157 (3)	0.083 (2)	0.145 (3)	−0.0318 (19)	0.088 (2)	0.0334 (19)
O3	0.0725 (16)	0.114 (2)	0.0840 (18)	−0.0154 (15)	0.0420 (14)	0.0119 (16)
O4	0.0380 (9)	0.0412 (10)	0.0561 (11)	−0.0026 (8)	0.0067 (8)	−0.0015 (9)
O5	0.0960 (17)	0.0644 (15)	0.0839 (16)	−0.0114 (13)	0.0483 (13)	−0.0033 (13)
O6	0.0788 (15)	0.0641 (14)	0.0879 (17)	0.0151 (12)	0.0367 (13)	−0.0108 (12)

### Geometric parameters (Å, °)

**Table:**

Ni1—N1	2.168 (2)	C16—H16	0.9300
Ni1—N4	2.143 (2)	C17—C18	1.438 (4)
Ni1—N7	2.121 (2)	C17—C26	1.464 (3)
Ni1—N8	2.122 (2)	C18—O4	1.284 (3)
Ni1—O1	2.0116 (16)	C18—C19	1.443 (3)
Ni1—O4	2.0161 (17)	C19—C20	1.351 (4)
N1—N2	1.295 (3)	C19—H19	0.9300
N1—C11	1.441 (3)	C20—C21	1.429 (4)
N2—C1	1.362 (3)	C20—H20	0.9300
N3—O2	1.222 (4)	C21—C26	1.409 (4)
N3—O3	1.225 (4)	C22—C23	1.366 (5)
N3—C14	1.462 (3)	C22—C21	1.412 (4)
N4—N5	1.306 (3)	C22—H22	0.9300
N4—C27	1.434 (3)	C23—C24	1.390 (6)
N5—C17	1.364 (3)	C23—H23	0.9300
N6—O5	1.230 (3)	C24—C25	1.388 (4)
N6—C30	1.474 (3)	C24—H24	0.9300
N7—C33	1.333 (3)	C25—C26	1.398 (4)
N7—C37	1.348 (3)	C25—H25	0.9300
N8—C38	1.335 (3)	C27—C32	1.394 (3)
N8—C42	1.341 (3)	C27—C28	1.402 (3)
C1—C2	1.437 (3)	C28—C29	1.374 (3)
C1—C10	1.462 (3)	C28—H28	0.9300
C2—O1	1.281 (3)	C29—C30	1.389 (3)
C2—C3	1.447 (3)	C29—H29	0.9300
C3—C4	1.350 (4)	C30—C31	1.380 (3)
C3—H3	0.9300	C31—C32	1.381 (3)
C4—C5	1.433 (4)	C31—H31	0.9300
C4—H4	0.9300	C32—H32	0.9300
C5—C6	1.409 (4)	C33—C34	1.381 (4)
C5—C10	1.412 (4)	C33—H33	0.9300
C6—C7	1.363 (5)	C34—C35	1.379 (5)
C6—H6	0.9300	C34—H34	0.9300
C7—H7	0.9300	C35—C36	1.363 (5)
C8—C9	1.384 (4)	C35—H35	0.9300
C8—C7	1.393 (5)	C36—C37	1.365 (4)
C8—H8	0.9300	C36—H36	0.9300
C9—C10	1.405 (4)	C37—H37	0.9300
C9—H9	0.9300	C38—C39	1.382 (4)
C11—C12	1.388 (3)	C38—H38	0.9300
C11—C16	1.394 (3)	C39—C40	1.381 (4)
C12—C13	1.375 (4)	C39—H39	0.9300
C12—H12	0.9300	C40—C41	1.368 (4)
C13—C14	1.378 (4)	C40—H40	0.9300
C13—H13	0.9300	C41—C42	1.375 (4)
C14—C15	1.367 (4)	C41—H41	0.9300
C15—C16	1.382 (3)	C42—H42	0.9300
C15—H15	0.9300	O6—N6	1.222 (3)
			
N1—N2—C1	122.0 (2)	C8—C7—H7	120.2
N2—C1—C2	125.4 (2)	C8—C9—C10	120.9 (3)
N2—C1—C10	114.1 (2)	C8—C9—H9	119.5
N2—N1—C11	110.14 (18)	C10—C9—H9	119.5
N2—N1—Ni1	121.05 (14)	C9—C10—C5	118.1 (2)
N4—Ni1—N1	88.18 (7)	C9—C10—C1	122.2 (2)
N4—N5—C17	120.4 (2)	C5—C10—C1	119.7 (2)
N5—N4—C27	111.37 (19)	C12—C11—C16	118.7 (2)
N5—N4—Ni1	119.66 (15)	C12—C11—N1	122.3 (2)
N5—C17—C18	125.0 (2)	C16—C11—N1	119.0 (2)
N5—C17—C26	114.9 (2)	C13—C12—C11	120.6 (3)
N7—Ni1—N1	90.70 (8)	C13—C12—H12	119.7
N7—Ni1—N4	175.45 (7)	C11—C12—H12	119.7
N7—Ni1—N8	85.96 (8)	C12—C13—C14	119.4 (3)
N7—C37—C36	122.9 (3)	C12—C13—H13	120.3
N7—C37—H37	118.5	C14—C13—H13	120.3
N7—C33—C34	122.5 (3)	C15—C14—C13	121.6 (2)
N7—C33—H33	118.7	C15—C14—N3	119.2 (3)
N8—C42—C41	123.1 (3)	C13—C14—N3	119.2 (3)
N8—C42—H42	118.5	C14—C15—C16	118.9 (2)
N8—C38—C39	123.1 (3)	C14—C15—H15	120.5
N8—C38—H38	118.5	C16—C15—H15	120.5
N8—Ni1—N1	174.80 (8)	C15—C16—C11	120.8 (2)
N8—Ni1—N4	95.45 (8)	C15—C16—H16	119.6
O1—Ni1—N4	93.84 (7)	C11—C16—H16	119.6
O1—Ni1—O4	175.75 (7)	C18—C17—C26	120.0 (2)
O1—Ni1—N7	90.40 (8)	C17—C18—C19	117.1 (2)
O1—Ni1—N8	92.99 (7)	C20—C19—C18	121.7 (3)
O1—Ni1—N1	83.04 (7)	C20—C19—H19	119.2
O1—C2—C1	124.2 (2)	C18—C19—H19	119.2
O1—C2—C3	119.0 (2)	C19—C20—C21	122.0 (3)
O2—N3—O3	123.4 (3)	C19—C20—H20	119.0
O2—N3—C14	117.4 (3)	C21—C20—H20	119.0
O3—N3—C14	119.2 (3)	C26—C21—C22	119.5 (3)
O4—Ni1—N4	82.23 (8)	C26—C21—C20	119.2 (3)
O4—Ni1—N7	93.47 (8)	C22—C21—C20	121.3 (3)
O4—Ni1—N8	89.03 (7)	C25—C26—C21	118.7 (3)
O4—Ni1—N1	95.16 (7)	C25—C26—C17	122.0 (3)
O4—C18—C17	124.3 (2)	C21—C26—C17	119.1 (3)
O4—C18—C19	118.6 (2)	C32—C27—C28	118.7 (2)
O5—N6—C30	118.2 (2)	C32—C27—N4	118.0 (2)
O6—N6—O5	123.1 (2)	C28—C27—N4	123.2 (2)
O6—N6—C30	118.8 (2)	C29—C28—C27	120.6 (2)
C11—N1—Ni1	126.17 (14)	C29—C28—H28	119.7
C27—N4—Ni1	124.23 (15)	C27—C28—H28	119.7
C9—C8—C7	120.5 (3)	C28—C29—C30	119.3 (2)
C9—C8—H8	119.7	C28—C29—H29	120.4
C7—C8—H8	119.7	C30—C29—H29	120.4
C23—C22—C21	120.9 (4)	C31—C30—C29	121.3 (2)
C23—C22—H22	119.5	C31—C30—N6	118.8 (2)
C21—C22—H22	119.5	C29—C30—N6	119.7 (2)
C22—C23—C24	119.7 (3)	C30—C31—C32	119.0 (2)
C22—C23—H23	120.2	C30—C31—H31	120.5
C24—C23—H23	120.2	C32—C31—H31	120.5
C25—C24—C23	120.8 (4)	C31—C32—C27	121.0 (2)
C25—C24—H24	119.6	C31—C32—H32	119.5
C23—C24—H24	119.6	C27—C32—H32	119.5
C24—C25—C26	120.4 (3)	C34—C33—H33	118.7
C24—C25—H25	119.8	C35—C34—C33	118.6 (3)
C26—C25—H25	119.8	C35—C34—H34	120.7
C33—N7—C37	117.6 (2)	C33—C34—H34	120.7
C33—N7—Ni1	121.11 (17)	C36—C35—C34	119.2 (3)
C37—N7—Ni1	121.06 (19)	C36—C35—H35	120.4
C38—N8—C42	117.1 (2)	C34—C35—H35	120.4
C38—N8—Ni1	122.17 (17)	C35—C36—C37	119.1 (3)
C42—N8—Ni1	120.36 (17)	C35—C36—H36	120.5
C2—C1—C10	120.4 (2)	C37—C36—H36	120.5
C1—C2—C3	116.8 (2)	C36—C37—H37	118.5
C4—C3—C2	122.0 (3)	C39—C38—H38	118.5
C4—C3—H3	119.0	C40—C39—C38	118.8 (3)
C2—C3—H3	119.0	C40—C39—H39	120.6
C3—C4—C5	122.5 (2)	C38—C39—H39	120.6
C3—C4—H4	118.7	C41—C40—C39	118.5 (3)
C5—C4—H4	118.7	C41—C40—H40	120.8
C6—C5—C10	119.5 (3)	C39—C40—H40	120.8
C6—C5—C4	122.1 (3)	C40—C41—C42	119.4 (3)
C10—C5—C4	118.3 (2)	C40—C41—H41	120.3
C7—C6—C5	121.3 (3)	C42—C41—H41	120.3
C7—C6—H6	119.3	C41—C42—H42	118.5
C5—C6—H6	119.3	C2—O1—Ni1	120.00 (14)
C6—C7—C8	119.5 (3)	C18—O4—Ni1	118.64 (15)
C6—C7—H7	120.2		
			
C21—C22—C23—C24	0.8 (6)	O2—N3—C14—C13	−9.6 (4)
C22—C23—C24—C25	−1.6 (6)	O3—N3—C14—C13	170.8 (3)
C23—C24—C25—C26	1.1 (6)	C13—C14—C15—C16	−1.7 (4)
O1—Ni1—N1—N2	43.27 (17)	N3—C14—C15—C16	177.1 (2)
O4—Ni1—N1—N2	−132.86 (17)	C14—C15—C16—C11	0.1 (4)
N7—Ni1—N1—N2	133.59 (17)	C12—C11—C16—C15	1.4 (4)
N4—Ni1—N1—N2	−50.82 (17)	N1—C11—C16—C15	−177.6 (2)
O1—Ni1—N1—C11	−156.98 (18)	N4—N5—C17—C18	−14.8 (4)
O4—Ni1—N1—C11	26.89 (18)	N4—N5—C17—C26	169.2 (2)
N7—Ni1—N1—C11	−66.66 (18)	N5—C17—C18—O4	14.6 (4)
N4—Ni1—N1—C11	108.92 (18)	C26—C17—C18—O4	−169.7 (2)
C11—N1—N2—C1	176.8 (2)	N5—C17—C18—C19	−166.3 (2)
Ni1—N1—N2—C1	−20.5 (3)	C26—C17—C18—C19	9.4 (3)
O1—Ni1—N4—N5	−127.58 (17)	O4—C18—C19—C20	169.1 (3)
O4—Ni1—N4—N5	50.77 (17)	C17—C18—C19—C20	−10.0 (4)
N8—Ni1—N4—N5	139.04 (17)	C18—C19—C20—C21	2.9 (4)
N1—Ni1—N4—N5	−44.68 (17)	C23—C22—C21—C26	0.5 (5)
O1—Ni1—N4—C27	25.88 (19)	C23—C22—C21—C20	179.8 (3)
O4—Ni1—N4—C27	−155.77 (19)	C19—C20—C21—C26	5.0 (4)
N8—Ni1—N4—C27	−67.50 (19)	C19—C20—C21—C22	−174.3 (3)
N1—Ni1—N4—C27	108.78 (18)	C24—C25—C26—C21	0.3 (5)
C27—N4—N5—C17	176.1 (2)	C24—C25—C26—C17	−174.7 (3)
Ni1—N4—N5—C17	−27.2 (3)	C22—C21—C26—C25	−1.1 (4)
O1—Ni1—N7—C33	36.6 (2)	C20—C21—C26—C25	179.6 (3)
O4—Ni1—N7—C33	−141.64 (19)	C22—C21—C26—C17	174.0 (3)
N8—Ni1—N7—C33	129.6 (2)	C20—C21—C26—C17	−5.3 (4)
N1—Ni1—N7—C33	−46.4 (2)	N5—C17—C26—C25	−10.9 (4)
O1—Ni1—N7—C37	−149.05 (19)	C18—C17—C26—C25	172.9 (3)
O4—Ni1—N7—C37	32.70 (19)	N5—C17—C26—C21	174.1 (2)
N8—Ni1—N7—C37	−56.08 (19)	C18—C17—C26—C21	−2.0 (4)
N1—Ni1—N7—C37	127.91 (19)	N5—N4—C27—C32	−158.6 (2)
O1—Ni1—N8—C38	−147.84 (19)	Ni1—N4—C27—C32	46.0 (3)
O4—Ni1—N8—C38	28.4 (2)	N5—N4—C27—C28	25.2 (3)
N7—Ni1—N8—C38	122.0 (2)	Ni1—N4—C27—C28	−130.2 (2)
N4—Ni1—N8—C38	−53.7 (2)	C32—C27—C28—C29	−1.7 (4)
O1—Ni1—N8—C42	39.14 (19)	N4—C27—C28—C29	174.5 (2)
O4—Ni1—N8—C42	−144.60 (19)	C27—C28—C29—C30	1.2 (4)
N7—Ni1—N8—C42	−51.05 (19)	C28—C29—C30—C31	0.7 (4)
N4—Ni1—N8—C42	133.29 (18)	C28—C29—C30—N6	−175.9 (2)
N1—N2—C1—C2	−14.7 (4)	O6—N6—C30—C31	−176.6 (2)
N1—N2—C1—C10	169.5 (2)	O5—N6—C30—C31	3.7 (4)
N2—C1—C2—O1	9.0 (4)	O6—N6—C30—C29	0.0 (4)
C10—C1—C2—O1	−175.5 (2)	O5—N6—C30—C29	−179.6 (3)
N2—C1—C2—C3	−169.8 (2)	C29—C30—C31—C32	−1.9 (4)
C10—C1—C2—C3	5.7 (3)	N6—C30—C31—C32	174.7 (2)
O1—C2—C3—C4	174.7 (2)	C30—C31—C32—C27	1.3 (4)
C1—C2—C3—C4	−6.4 (4)	C28—C27—C32—C31	0.5 (4)
C2—C3—C4—C5	2.4 (4)	N4—C27—C32—C31	−175.9 (2)
C3—C4—C5—C6	−177.6 (3)	C37—N7—C33—C34	−1.2 (4)
C3—C4—C5—C10	2.4 (4)	Ni1—N7—C33—C34	173.4 (2)
C10—C5—C6—C7	0.2 (4)	N7—C33—C34—C35	1.0 (5)
C4—C5—C6—C7	−179.9 (3)	C33—C34—C35—C36	0.0 (5)
C5—C6—C7—C8	1.1 (5)	C34—C35—C36—C37	−0.8 (5)
C9—C8—C7—C6	−1.7 (5)	C33—N7—C37—C36	0.3 (4)
C7—C8—C9—C10	1.1 (5)	Ni1—N7—C37—C36	−174.2 (2)
C8—C9—C10—C5	0.1 (4)	C35—C36—C37—N7	0.7 (5)
C8—C9—C10—C1	−177.7 (3)	C42—N8—C38—C39	1.0 (4)
C6—C5—C10—C9	−0.8 (4)	Ni1—N8—C38—C39	−172.3 (2)
C4—C5—C10—C9	179.3 (2)	N8—C38—C39—C40	0.6 (4)
C6—C5—C10—C1	177.1 (2)	C38—C39—C40—C41	−1.3 (4)
C4—C5—C10—C1	−2.9 (4)	C39—C40—C41—C42	0.5 (4)
N2—C1—C10—C9	−7.5 (4)	C38—N8—C42—C41	−1.8 (4)
C2—C1—C10—C9	176.5 (2)	Ni1—N8—C42—C41	171.5 (2)
N2—C1—C10—C5	174.8 (2)	C40—C41—C42—N8	1.1 (4)
C2—C1—C10—C5	−1.2 (3)	C1—C2—O1—Ni1	34.0 (3)
N2—N1—C11—C12	17.2 (3)	C3—C2—O1—Ni1	−147.22 (18)
Ni1—N1—C11—C12	−144.4 (2)	N7—Ni1—O1—C2	−138.84 (17)
N2—N1—C11—C16	−163.9 (2)	N8—Ni1—O1—C2	135.18 (17)
Ni1—N1—C11—C16	34.5 (3)	N4—Ni1—O1—C2	39.51 (18)
C16—C11—C12—C13	−1.4 (4)	N1—Ni1—O1—C2	−48.18 (17)
N1—C11—C12—C13	177.5 (2)	C17—C18—O4—Ni1	30.8 (3)
C11—C12—C13—C14	−0.1 (4)	C19—C18—O4—Ni1	−148.24 (19)
C12—C13—C14—C15	1.7 (4)	N7—Ni1—O4—C18	128.08 (18)
C12—C13—C14—N3	−177.1 (3)	N8—Ni1—O4—C18	−146.03 (18)
O2—N3—C14—C15	171.5 (3)	N4—Ni1—O4—C18	−50.39 (18)
O3—N3—C14—C15	−8.0 (4)	N1—Ni1—O4—C18	37.06 (18)

### Hydrogen-bond geometry (Å, °)

**Table:**

*D*—H···*A*	*D*—H	H···*A*	*D*···*A*	*D*—H···*A*
C36—H36···O2^i^	0.93	2.38	3.124 (5)	137
C41—H41···O6^ii^	0.93	2.53	3.205 (4)	130

Symmetry codes: (i) −*x*+2, *y*−1/2, −*z*−1/2; (ii) −*x*+1, *y*−1/2, −*z*+1/2.
